# Supplementary material for: Net decrease in spine-surface GluA1-containing AMPA receptors after post-learning sleep in the adult mouse cortex
Source: Nat Commun. 2021 May 17;12:2881. doi: 10.1038/s41467-021-23156-2 (PMC8129120; doi:10.1038/s41467-021-23156-2)
Supplement: Supplementary file 1 — Supplementary Information [file 41467_2021_23156_MOESM1_ESM.pdf]

Net decrease in spine-surface GluA1-containing AMPA receptors after post-learning sleep in the adult mouse cortex

Daisuke Miyamoto, William Marshall, Giulio Tononi and Chiara Cirelli[1]

This file contains:

Supplementary figures 1-7

Supplementary tables 1-5

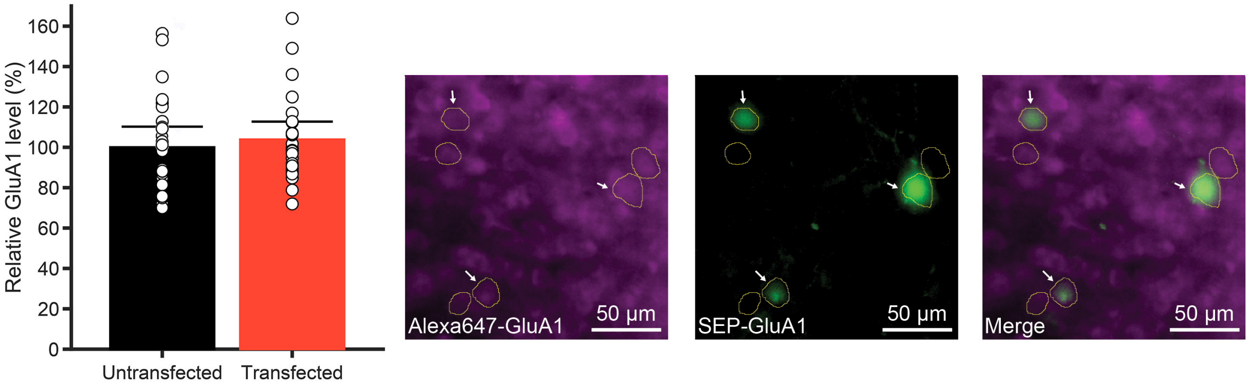

Supplementary Figure 1. Left, quantification of relative levels of GluA1 expression in transfected (red) and untransfected (black) neurons (28 cells/ group, 6 sections in 2 mice; mean ± SEM). GluA1 expression level in untransfected neurons are normalized to 100%; $p = 0.4461$, Wilcoxon rank sum test, two-sided. Right, examples of immunostaining of Alexa647-GluA1 intensity and GFP to reveal SEP-GluA1 in brain slices of electroporated pups. The level of total GluA1 is similar between transfected and untransfected neurons.

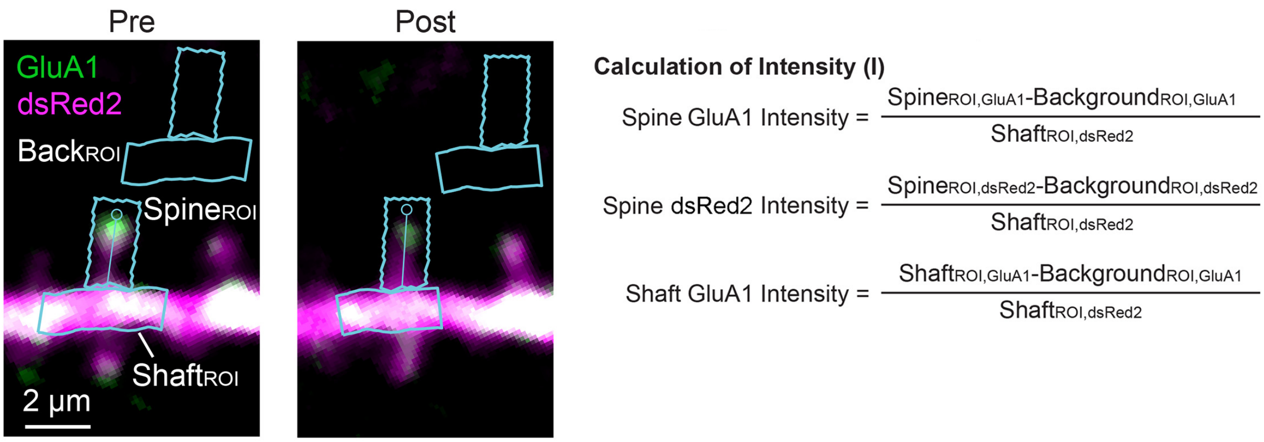

Supplementary Figure 2. Representative examples of the region of interest (ROI) used to calculate the expression levels of GluA1 and dsRed2 in the spine and shaft (experiments were run in 12 mice). Back = background.

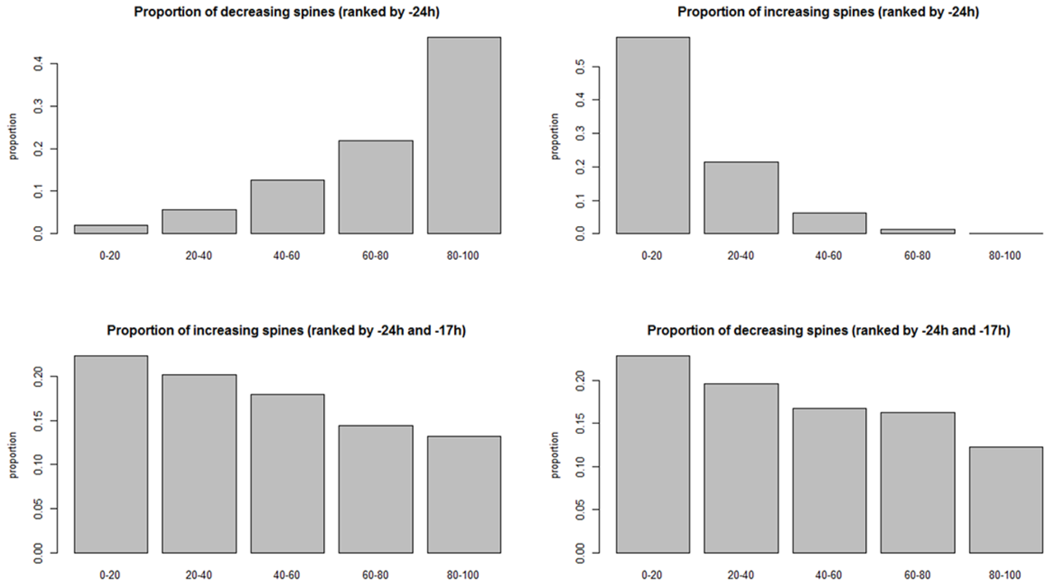

Supplementary Figure 3. Results of the simulation study showing that using two time points for ranking is sufficient to overcome regression to the mean (RTTM) effects in the current analysis. The intensities at -24h and -17h were simulated as independent and normally distributed, with common mean and variance. For each spine, we computed the normalized difference (ND) between -24h and -17h and classified the spine as having increased if ND > 0.15 or decreased if ND < -0.15. Spines were then ranked into quintiles (based on either the intensity at -24h, or the average intensity of -24h and -17h). As shown in the plots below, there is a clear RTTM effect when spines were ranked by -24h alone: small spines are more likely to increase, and big spines are more likely to decrease. However, when spines are ranked into quintiles based on the average of -24h and -17h, then the RTTM effect disappears. There is still a tendency for small spines to change more than large spines, but this is not an effect of RTTM, since it occurs for both increasing and decreasing spines. Rather, this result is expected if the effect is consistent for all spines: small spines change more often because they are smaller to begin with, and thus require a smaller absolute change in order to cross the ND threshold.

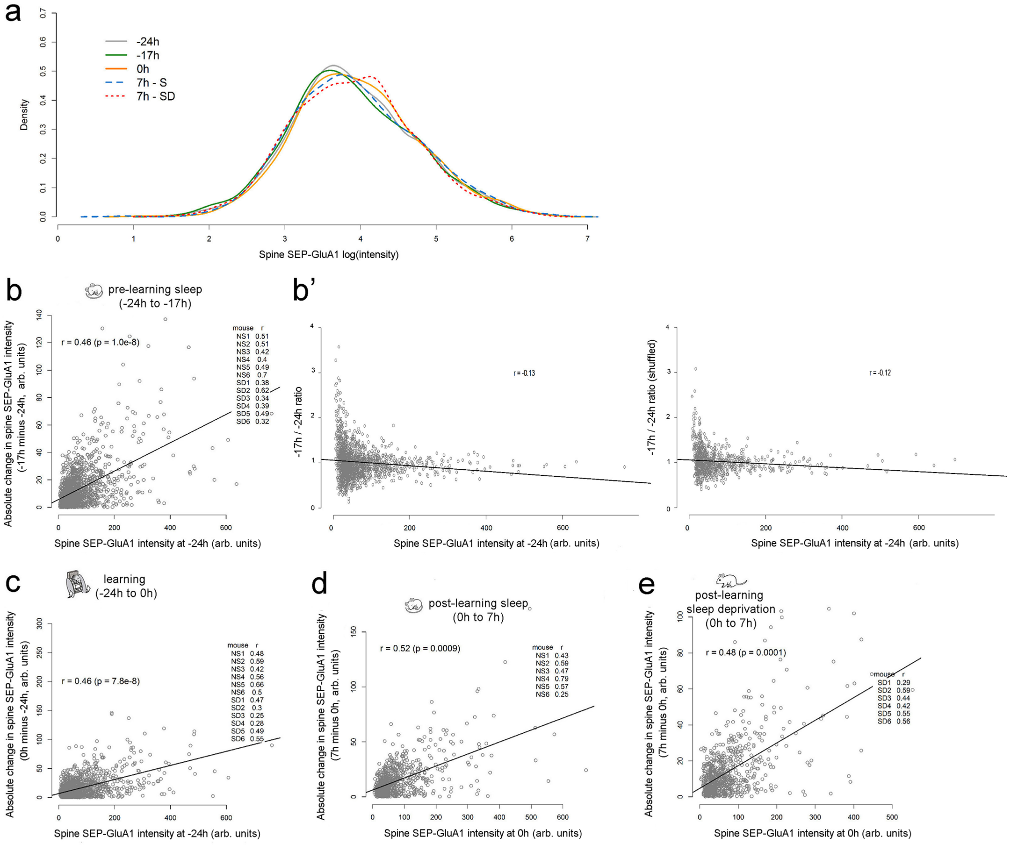

Supplementary Figure 4. Log-normal distributions of the intensities of spine SEP-GluA1 and their relationship with the changes in spine SEP-GluA1 expression. (a) Log-normal distributions of the intensities of spine SEP-GluA1 at each time point (1530 spines in 12 mice for -24h, -17h, 0h; 779 spines in 6 S mice; 751 spines in 6 SD mice). S, sleep; SD, sleep deprivation. **(b-e)** Correlations between the baseline intensities of spine SEP-GluA1 and the change in spine SEP-GluA1 intensity after pre-learning sleep. All correlations were computed per mouse and then the per-mouse correlations were averaged together to get an overall estimate; **(b),** learning **(c),** post-learning sleep **(d)**, post-learning sleep deprivation **(e)**. For **b**, **c**, **d**, **e**, we test the hypothesis that the average correlation (per mouse) is zero using a two-sided one-sample *t* test. **(b')** left, a negative correlation between wake levels of spine SEP-GluA1 (-24h) and the ratio of sleep/waking expression (-17h / -24h) is present in our data, consistent with the result in [22]. This result should not be interpreted as evidence that the sleep-dependent decline in SEP-GluA1 expression occurs mainly or exclusively in the largest spines, because a similar negative correlation is present when the sleep/waking labels are shuffled (b', right panel). This is because "waking" appears in the denominator of the ratio, thus an increase in waking necessarily leads to a decrease in the sleep/waking ratio. GluA1 and dsRed2 intensities were calculated as detailed in Supplementary Figure 2. Arb. units = arbitrary units. Source data are provided as a Source Data file.

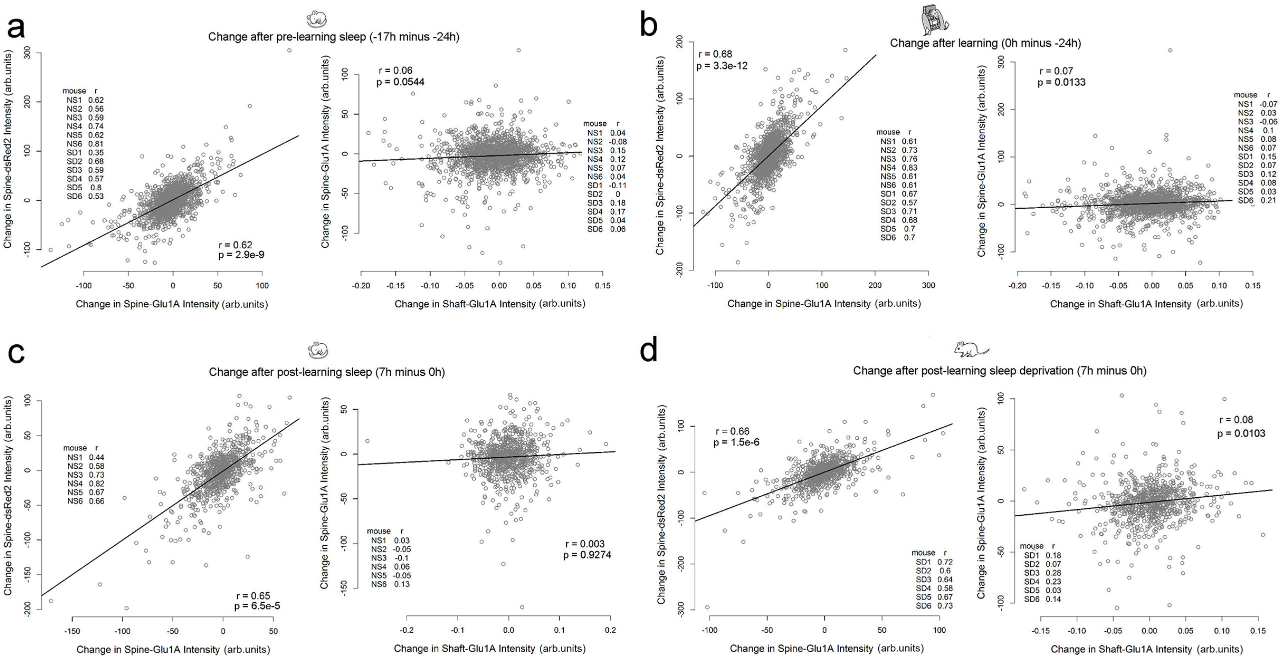

Supplementary Figure 5. Correlations between the changes in spine SEP-GluA1 intensity and the changes in spine dsRed2 intensity or in shaft SEP-GluA1 intensity. All correlations were computed per mouse and then the per-mouse correlations were averaged together to get an overall estimate. **(a)** Change after pre-learning sleep (-17h minus -24h). **(b)** Change after learning (0h minus -24h). **(c)** Change after post-learning sleep (7h minus 0h). **(d)** Change after post-learning sleep deprivation (7h minus 0h). GluA1 and dsRed2 intensities were calculated as detailed in Supplementary Figure 2. Arb. units = arbitrary units. For **a**, **b**, **c**, **d**, we test the hypothesis that the average correlation (per mouse) is zero using a two-sided one-sample *t* test. Source data are provided as a Source Data file.

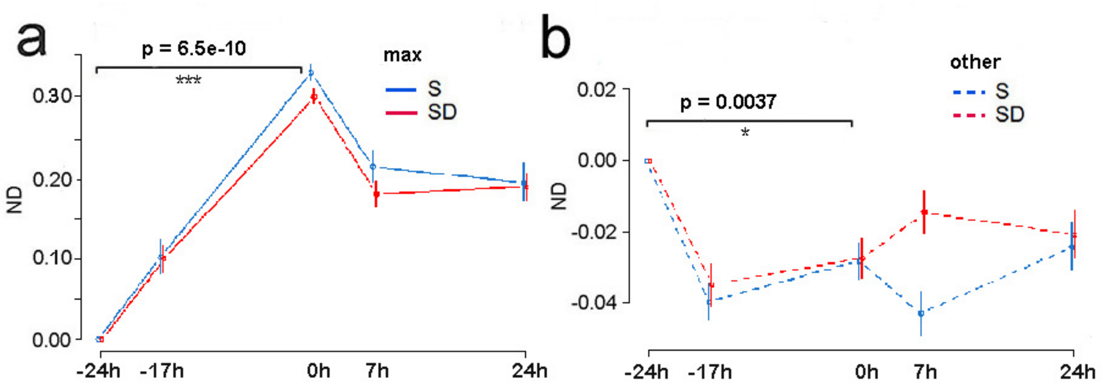

Supplementary Figure 6. Normalized difference (ND, mean ± SEM) of spine SEP-GluA1 expression relative to -24h in the max spines (**a**) and the other spines (**b**). Statistical values are shown between -24h and 0h, because this is the interval that was used to define and rank max spines and other spines. S, sleep (*n* = 6 mice); SD, sleep deprivation (*n* = 6 mice). N of spines: S, max = 96; S, other = 683; SD, max = 111; SD, other = 640. Max spines as % of all spines = 13.5 ± 4.1% (207/1530; mean ± std; % of max spines per mouse, range = 6.3% - 20%). For **a**, **b**, *p* values reported are based on two-sided likelihood ratio test. Source data are provided as a Source Data file.

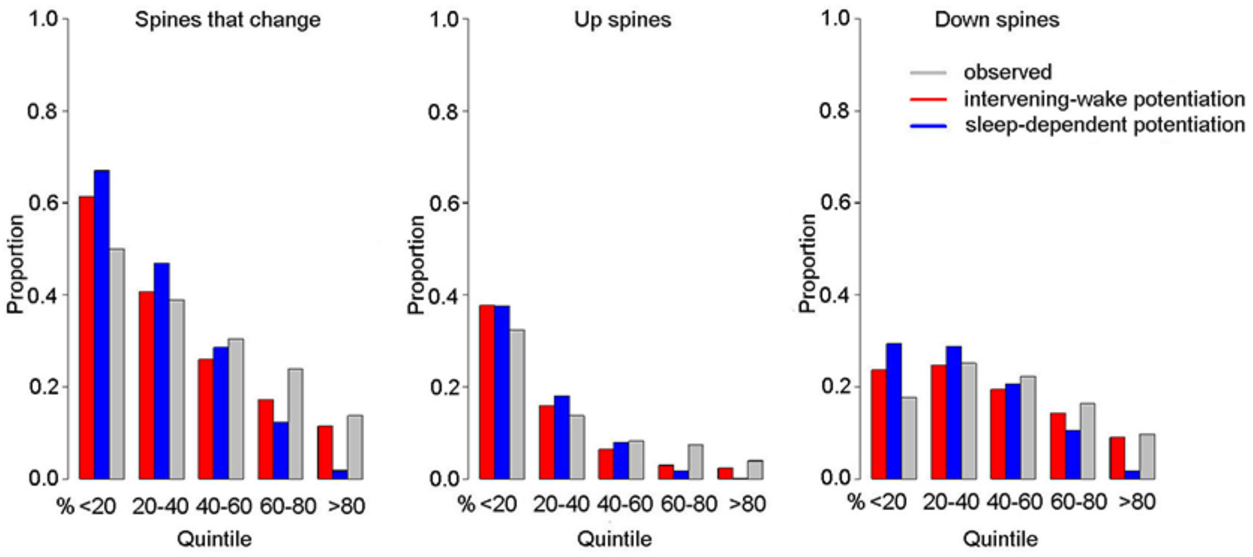

Supplementary Figure 7. Comparison between the ability of two models (intervening-wake potentiation model and sleep-dependent potentiation model) to match the observed number of spines changing in each quintile (all, up, down) using a normalized difference of ± 0.15.

Supplementary Table 1. Detailed statistical analysis of performance data using two-sided paired-sample t-tests and two-sided independent-sample t-tests as appropriate.

**Mean difference**	**Standard deviation**	**95% CI**	**t**	**df**	**p-value**	**Cohen's d**
Average performance in first three trials vs. last three trials of the first session (paired t-test, *n* = 12 mice)						
-5.670	3.983	[-8.20, -3,14]	-4.931	11	0.0004	1.42
Average performance in first three trials of the second session (S vs. SD; independent sample t-test; 6 mice / group)						
2.422	1.572	[0.40, 4.44]	2.668	10	0.0236	1.54

Supplementary Table 2. Detailed statistical analysis for all correlations. Correlations computed for each mouse and then a two-sided, one-sample t-test is used to test the hypothesis that the average correlation is zero. For (*) comparisons, there is only one observation per mouse and Fisher's transformation is used to test the hypothesis that correlation is zero.

Variables	Mean correlation	Standard deviation	95% CI	t	df	p-value
**Baseline correlations (-24h)**						
Spine GluA1 vs. spine dsRed2	0.935	0.038	[0.91, 0.96]	84.14	11	< 2.2e-16
Spine GluA1 vs. shaft GluA1	0.178	0.127	[0.10, 0.26]	4.83	11	0.0005
**Change correlations (-24h to -17h)**						
Spine GluA1 vs. spine dsRed2	0.622	0.126	[0.54, 0.70]	17.06	11	2.9e-9
Spine GluA1 vs. shaft GluA1	0.057	0.091	[-0.01, 0.12]	2.15	11	0.0544
**Change correlations (-24h to 0h)**						
Spine GluA1 vs. spine dsRed2	0.682	0.074	[0.64, 0.73]	31.98	11	3.3e-12
Spine GluA1 vs. shaft GluA1	0.068	0.079	[0.02, 0.12]	2.948	11	0.0133
**Change correlations (0h to 7h; S)**						
Spine GluA1 vs. spine dsRed2	0.650	0.130	[0.51, 0.79]	12.22	5	6.5e-5
Spine GluA1 vs. shaft GluA1	0.003	0.085	[-0.09, 0.09]	0.10	5	0.9274
**Change correlations (0h to 7h; SD)**						
Spine GluA1 vs. spine dsRed2	0.657	0.062	[0.59, 0.72]	26.14	5	1.5e-6
Spine GluA1 vs. shaft GluA1	0.155	0.095	[0.06, 0.25]	4.00	5	0.0103
**Scaling correlations (-24h vs. absolute change)**						
Spine GluA1 (-24h to -17h)	0.464	0.113	[0.39, 0.54]	14.24	11	2.0e-8
Spine GluA1 (-24h to 0h)	0.463	0.128	[0.38, 0.54]	12.47	11	7.8e-8
Spine GluA1 (0h to 7h; S)	0.517	0.181	[0.33, 0.71]	6.99	5	0.0009
Spine GluA1 (0h to 7h; SD)	0.475	0.114	[0.36, 0.59]	10.24	5	0.0002
**Correlation of baseline (-24h) with change ratio (-17h / -24h)**						
Spine GluA1	-0.137	0.087	[-0.19, - 0.08]	-5.46	11	0.0002

**(*) Correlation with task performance (first 3 trials of second session)**						
Max/other interaction $(7h - 0h)_{max} - (7h - 0h)_{other}$	0.1421		[-0.47, 0.66]	0.45	10	0.6594
ND (7h relative to 0h; all spines)	-0.6406		[-0.89, -0.11]	-2.64	10	0.0248
ND (7h relative to 0h; other only)	-0.5946		[-0.87, -0.03]	-2.34	10	0.0414
ND (7h relative to 0h; max only)	-0.3924		[-0.79, 0.23]	-1.35	10	0.2071

Supplementary Table 3. Detailed statistical analysis for all linear mixed effect models. Two-sided likelihood ratio tests for main effects and interactions, and then two-sided *z* tests for post-hoc comparisons. Post-hoc comparisons are corrected for multiple comparisons. Note: the Sleep factor has levels before sleep (-24h and 0h) and after sleep (-17h and 7h), and the Training factor has levels before training (-24h, -17h) and after training (0h, 7h).

**Likelihood Ratio Tests**					
**Effect**	**χ^2**	**df**	**p-value**	**Cohen's f^2**	
Time (-24h, -17h, 0h)	74.612	2	< 2.2e-16	0.025	
Time (0h, 7h) * Condition	5.108	1	0.0238	0.003	
Max * Condition	4.058	1	0.0440	0.004	
Max * Condition (-17h to 0h and -24h to 0h)	4.554	1	0.0328	0.004	
Max * Condition (-17h to 0h; not -24h to 0h)	0.040	1	0.8411	0.00006	
Max * Time (7h, 24h)	0.6605	1	0.4164	0.0004	
Sleep * Training	0.1317	1	0.7167	0.00003	
**Post-hoc Tests**					
**Contrast**	**mean**	**standard deviation**	**z**	**p-value**	**95% CI**
-24h to -17h	-0.132	0.030	-4.39	3.6e-6	[-0.202, -0.062]
-24h to 0h	0.129	0.030	4.30	4.5e-5	[0.059, 0.200]
-17h to 0h	0.261	0.030	8.69	< 1e-16	[0.332, 0.191]
0h to 7h (S)	-0.193	0.040	-4.87	< 2.2e-6	[-0.281, -0.104]
0h to 7h (SD)	-0.065	0.040	-1.61	0.202	[-0.155, 0.025]
-17h to 7h (Max)	0.8432	0.2925	2.883	0.0044	[0.270, 1.416]
-17h to 7h (Other)	0.2431	0.1692	1.437	0.1510	[-0.089, 0.575]
Learning * Condition effect	0.3239	0.160	2.022	0.0440	[0.010, 0.638]

Supplementary Table 4. Detailed statistical analysis of the percentage of the difference between the proportion of spines that increase and the proportion of spines that decrease (per mouse). Spines are defined as increase or decreasing using ND. All tests are two-sided paired t-tests.

ND Threshold	Mean difference	Standard deviation	95% CI	t	df	p-value	Cohen's d
**Changes from -24h to -17h**							
0.1	-0.077	0.076	[-0.125, -0.029]	-3.52	11	0.0048	1.02
0.15	-0.051	0.058	[-0.088, -0.014]	-3.01	11	0.0120	0.87
0.2	-0.032	0.048	[-0.063, -0.002]	-2.35	11	0.0386	0.67
**Changes from -24h to 0h**							
0.1	0.061	0.094	[0.001, 0.120]	2.23	11	0.0476	0.64
0.15	0.061	0.068	[0.018, 0.104]	3.13	11	0.0097	0.90
0.2	0.046	0.033	[0.025, 0.067]	4.74	11	0.0006	1.37
**Changes from 0h to 7h (S condition)**							
0.1	-0.103	0.095	[-0.203, -0.003]	-2.66	5	0.0450	1.08
0.15	-0.081	0.069	[-0.153, -0.009]	-2.87	5	0.0349	1.17
0.2	-0.047	0.053	[-0.103, 0.008]	-2.18	5	0.0810	0.89
**Changes from 0h to 7h (SD condition)**							
0.1	-0.021	0.086	[-0.112, 0.069]	-0.607	5	0.5704	0.25
0.15	-0.021	0.048	[-0.072, 0.029]	-1.093	5	0.3242	0.45
0.2	-0.007	0.039	[-0.048, -0.034]	-0.444	5	0.6754	0.18

Supplementary Table 5. Parameter estimates and relative errors for the two models of sleep for spine SEP-Glu1A intensity.

	**Intervening-wake potentiation**	**Sleep-dependent potentiation**
**Parameters**		
Standard deviation (independent noise)	8.2	13.45
Standard deviation (size-dependent noise)	14.16	-
downscaling proportion	0.3	0.6
downscaling magnitude	0.915	0.925
upscaling proportion	-	0.2
upscaling magnitude	-	1.04
**Relative Errors**		
-17h mean	-0.21%	0.37%
-17h standard deviation	1.72%	0.77%
(-24h, -17h) correlation	0.87%	1.68%
proportion up	-0.35%	-0.49%
proportion down	-0.08%	0.08%
**Total Relative Error**	3.26%	3.39%
