# Supplementary material for: Net decrease in spine-surface GluA1-containing AMPA receptors after post-learning sleep in the adult mouse cortex
Source: Nat Commun. 2021 May 17;12:2881. doi: 10.1038/s41467-021-23156-2 (PMC8129120; doi:10.1038/s41467-021-23156-2)
Supplement: Supplementary file 3 — Reporting Summary [file 41467_2021_23156_MOESM3_ESM.pdf]

nature research

Corresponding author(s): Chiara Cirelli

Last updated by author(s): Mar 29, 2021

Reporting Summary

Nature Research wishes to improve the reproducibility of the work that we publish. This form provides structure for consistency and transparency in reporting. For further information on Nature Research policies, see our Editorial Policies and the Editorial Policy Checklist.

Statistics

For all statistical analyses, confirm that the following items are present in the figure legend, table legend, main text, or Methods section.

n/a	Confirmed	
☐	☒	The exact sample size (n) for each experimental group/condition, given as a discrete number and unit of measurement
☐	☒	A statement on whether measurements were taken from distinct samples or whether the same sample was measured repeatedly
☐	☒	The statistical test(s) used AND whether they are one- or two-sided<br>*Only common tests should be described solely by name; describe more complex techniques in the Methods section.*
☐	☒	A description of all covariates tested
☐	☒	A description of any assumptions or corrections, such as tests of normality and adjustment for multiple comparisons
☐	☒	A full description of the statistical parameters including central tendency (e.g. means) or other basic estimates (e.g. regression coefficient) AND variation (e.g. standard deviation) or associated estimates of uncertainty (e.g. confidence intervals)
☐	☒	For null hypothesis testing, the test statistic (e.g. F, t, r) with confidence intervals, effect sizes, degrees of freedom and P value noted<br>*Give P values as exact values whenever suitable.*
☒	☐	For Bayesian analysis, information on the choice of priors and Markov chain Monte Carlo settings
☒	☐	For hierarchical and complex designs, identification of the appropriate level for tests and full reporting of outcomes
☐	☒	Estimates of effect sizes (e.g. Cohen's d, Pearson's r), indicating how they were calculated

Our web collection on statistics for biologists contains articles on many of the points above.

Software and code

Policy information about availability of computer code

Data collection	Motor activity to assess sleep/wake behavior was quantified by custom-made video-based motion detection algorithms written in Matlab.<br>In vivo imaging was performed with a two-photon microscope (Ultima, Prairie Technologies/Bruker, Middleton, WI) and a Ti:Sapphire laser (Cameleon Coherent, Coherent Inc., Santa Clara, CA) tuned to the excitation wavelength of 910 nm. Images were taken using a water immersion 60X objective (0.8 numerical aperture, Olympus, LUMPlanFI/IR, Tokyo, Japan).
Data analysis	Image stacks were aligned and registered using the MultiStackReg plugin of ImageJ . Manually marked spine heads using the structural dsRed2 channel were automatically connected to each traced dendrite, using a custom-written Map Manager software (http://robertcudmore.org/) on IGOR Pro (Wavemetrics, Lake Oswego, OR), kindly shared by Dr. Robert Cudmore (University of California, Davis).<br>In Figure 1a, a representative stack was 3D-reconstructed using the 3D viewer plugin 66 in the Fiji image analysis software.<br>Statistical analysis of SEP-GluA1 intensities was performed using linear mixed effect (LME) models. Maximum likelihood estimation of model parameters was performed in R using the lme4 package. For significant effects, post-hoc tests with p-values corrected for multiple comparisons were performed using the glht() function in the multcomp package.

For manuscripts utilizing custom algorithms or software that are central to the research but not yet described in published literature, software must be made available to editors and reviewers. We strongly encourage code deposition in a community repository (e.g. GitHub). See the Nature Research guidelines for submitting code & software for further information.

Data

Policy information about availability of data

All manuscripts must include a data availability statement. This statement should provide the following information, where applicable:

- Accession codes, unique identifiers, or web links for publicly available datasets
- A list of figures that have associated raw data
- A description of any restrictions on data availability

The source data for the figures in this article are provided. All relevant data are available from the authors upon request.

Field-specific reporting

Please select the one below that is the best fit for your research. If you are not sure, read the appropriate sections before making your selection.

[x] Life sciences   [ ] Behavioural & social sciences   [ ] Ecological, evolutionary & environmental sciences

For a reference copy of the document with all sections, see nature.com/documents/nr-reporting-summary-flat.pdf

Life sciences study design

All studies must disclose on these points even when the disclosure is negative.

Sample size	Sample size was determined based on past experience with repeated two-photon imaging (Maret et al., Nature Neuroscience 2011) and pilot experiments.
Data exclusions	13 transfected mice with well consolidated 24h sleep/waking cycles were used. One mouse was discarded before the start of the first session of two-photon imaging due to very weak dendritic fluorescence. All remaining 12 mice were used and completed all steps of the experiment.
Replication	All replication attempts were successful (12 mice).
Randomization	Mice were randomly assigned to experimental groups. Mice were run in separate experiments, alternating between the two experimental conditions (sleep, sleep deprivation) as much as possible.
Blinding	During data collection blinding was not possible, but image analysis of spines and shafts was performed blind to experimental conditions (sleep or SD, motor performance, and imaging sessions).

Reporting for specific materials, systems and methods

We require information from authors about some types of materials, experimental systems and methods used in many studies. Here, indicate whether each material, system or method listed is relevant to your study. If you are not sure if a list item applies to your research, read the appropriate section before selecting a response.

Materials & experimental systems

n/a	Involved in the study	
[ ]	[x]	Antibodies
[x]	[ ]	Eukaryotic cell lines
[x]	[ ]	Palaeontology and archaeology
[ ]	[x]	Animals and other organisms
[x]	[ ]	Human research participants
[x]	[ ]	Clinical data
[x]	[ ]	Dual use research of concern

Methods

n/a	Involved in the study	
[x]	[ ]	ChIP-seq
[x]	[ ]	Flow cytometry
[x]	[ ]	MRI-based neuroimaging

Antibodies

Antibodies used	anti-GluA1 antibody conjugated to Alexa Fluor 647 (1:50, Santa Cruz Biotechnology, sc-55509 AF647)
Validation	mouse monoclonal IgG1 (kappa light chain) validated using Western blot analysis of GluR-1 expression in mouse brain (A), rat brain (B) and rat cerebellum (C) tissue extracts; 18 citations.

Animals and other organisms

Policy information about studies involving animals; ARRIVE guidelines recommended for reporting animal research

Laboratory animals	Embryos from timed pregnant C57BL/6 mice (Jackson Laboratory) were transfected by IUE at embryonic day 14.5 (E14.5), in order to restrict expression to cortical layers 2/3 excitatory neurons of primary motor cortex. Adult mice (3-4 months old, both sexes) with expression in primary motor cortex were used for the experiments. They were maintained on a 12 h light/12 h dark cycle with food and water available ad libitum (21–23 °C, 30–40% relative humidity).
Wild animals	No wild animals were used in the study.
Field-collected samples	No field collected samples were used in the study.
Ethics oversight	All animal procedures and experimental protocols followed the National Institutes of Health Guide for the Care and Use of Laboratory Animals and were approved by the licensing committee. Animal facilities were reviewed and approved by the institutional animal care and use committee (IACUC) of the University of Wisconsin-Madison, and were inspected and accredited by the association for assessment and accreditation of laboratory animal care (AAALAC).

Note that full information on the approval of the study protocol must also be provided in the manuscript.
